# Mice Condition Cephalic Insulin Responses to the Flavor of Different Laboratory Chows

**DOI:** 10.3390/nu17243880

**Published:** 2025-12-12

**Authors:** Laura Mittelman, Natalie Ashkar, Fatima Khwaja, Clara Resnick, John I. Glendinning

**Affiliations:** 1 Department of Neuroscience & Behavior, Barnard College, Columbia University, New York, NY 10027, USA; lauramittelman5@gmail.com (L.M.); cjr2208@barnard.edu (C.R.); 2 Department of Biology, Barnard College, Columbia University, New York, NY 10027, USA; fatimabkhwaja@gmail.com

**Keywords:** insulin, chewing, cephalic-phase response, mouse, olfaction

## Abstract

**Background/Objectives**: Cephalic-phase insulin responses (CPIRs) are characterized as the pre-absorptive release of insulin triggered by sensory stimuli associated with eating or drinking. CPIRs are beneficial because they reduce postprandial elevations in blood glucose. **Methods**: We investigated whether the flavor of two different types of laboratory chow elicits a CPIR in mice (C57BL/6). **Results**: First, we tried unsuccessfully to replicate a prior report that a single bite from a familiar chow pellet elicits a CPIR. Second, we determined that a minimum of 15 s of chewing on a familiar chow was necessary to elicit a CPIR. Third, we asked whether the chow-induced CPIR required prior exposure to the same chow. We tested the responses to a standard and a purified chow, which had similar macronutrient compositions. Mice raised on standard chow generated a CPIR to standard chow but not the novel purified chow. After 4 (but not 2) weeks of exposure to the purified chow, however, the mice generated a CPIR to it. Likewise, mice raised on purified chow generated a CPIR to purified chow but not to the novel standard chow. After 2–4 weeks of exposure to standard chow, however, the mice conditioned a CPIR to it. It follows that mice had to condition CPIRs to each type of chow. Fourth, we established that olfactory impairment eliminated the conditioned CPIR to standard chow (when it was familiar), but not to purified chow (when it was familiar). **Conclusions**: The flavor of familiar chow reliably triggered a CPIR in mice, but this response had to be conditioned through dietary exposure. Olfaction was a critical component of the conditioned stimulus for standard but not purified chow.

## 1. Introduction

The mere flavor of foods or beverages can trigger insulin secretion in humans and other mammals [1,2,3,4,5,6]. This sensory-mediated hormonal response, referred to as a cephalic-phase insulin response (CPIR), is initiated by orosensenory stimulation, which causes parasympathetic stimulation of beta cells in the pancreas [7]. CPIRs play a critical physiological role in limiting postprandial elevations in blood glucose during and after a meal [6] through two complementary mechanisms: enhancing pancreatic β-cell sensitivity to glucose, thereby promoting more rapid and robust insulin release [8,9], and suppressing hepatic glucose production [10]. The net effect is improved regulation of blood glucose.

Despite many advances in our understanding of CPIR over the past five decades, little is known about the extent to which food-elicited CPIRs reflect an unlearned endocrine reflex versus a conditioned response [3,5,6]. This issue cannot be addressed easily in humans due to substantial interindividual variability in dietary histories, making it difficult to identify novel flavors for conditioning trials. The diet of laboratory rodents, by contrast, is typically limited to water and a single type of chow. This makes rodents an excellent model system for exploring the etiology of food-elicited CPIRs.

There are several lines of evidence that CPIRs are subject to conditioning in rodents. On the one hand, oral stimulation with novel glucose or saccharin solutions elicited a robust CPIR in rats, but when consumption of either of these solutions was paired with an aversive postoral experience (i.e., an intraperitoneal injection of lithium chloride), the CPIR was suppressed [11,12]. On the other hand, the time of day, the sound of a food-hopper door opening, and the presence of a strong odor all served as effective conditioned stimuli when they reliably predicted the presentation of food in rats [13,14] and mice [15]. There is also a report of mice conditioning a CPIR to the flavor of a 32% maltodextrin solution after five days of dietary exposure to the same solution [16].

A knowledge gap exists regarding cephalic phase responses to foods. Despite reports that the flavor of familiar laboratory chows can trigger cephalic insulin responses in rats [1,17] and mice [18], the investigators did not determine whether conditioning was required. Here, we examined the necessity of conditioning to chow-induced CPIRs in mice. To this end, we conducted five experiments. In Experiment 1, we tried to replicate a prior report that a single bite from a familiar chow pellet is sufficient to elicit CPIR in B6 mice [18]. Because our replication efforts were unsuccessful, Experiment 2 sought to determine a more reliable method for eliciting CPIR with familiar chow pellets. Experiment 3 examined the necessity of conditioning to chow-induced CPIRs. Experiment 4 compared ingestion rates of the two types of chow to determine whether differences in chow consistency could explain why the conditioned CPIR failed to cross-generalize between the chows. Experiment 5 asked how impairing olfaction affected the conditioned CPIR to both standard and purified chows.

## 2. Methods

### 2.1. Animals, Housing Conditions and Protocol Approvals

C57BL/6 (B6; stock no. 000664) mice came from a colony derived from individuals purchased at Jackson Laboratories (Bar Harbor, ME, USA). Approximately equal numbers of males and females were used in each treatment group. Because secondary analyses revealed no effect of sex on any of the outcome measures, we combined data from both sexes.

We tested a total of 211 mice (106 males; 105 females). In all experiments, the mouse was the experimental unit. Sample sizes for each experiment and treatment level are provided in the figure legends. In all experiments, mice were randomly assigned by litter to treatment groups. At the start of the experiment, the mice were 7–9 weeks old and weighed 20–28 g. They were group-housed in caging racks at Columbia University under temperature-controlled conditions in a room with a 12:12 h light-dark cycle. At the start of each experiment, the mice were housed individually. They had unlimited access to tap water and standard chow, unless indicated otherwise, and obtained water from sipper spouts attached to water bottles. At the end of each experiment, the mice were euthanized with carbon dioxide inhalation.

All protocols were approved by the Institutional Animal Care and Use Committee of Columbia University. They were conducted in accordance with the National Institutes of Health guidelines for the care and use of laboratory animals.

### 2.2. Types of Chow

We offered the mice two types of chow. The standard chow (Laboratory rodent diet, 5001, LabDiet, St. Louis, MO, USA) had an energy density of 3.4 kcal/g. The macronutrient breakdown by % kcal was 57.5% carbohydrate, 13.6% fat and 28.9% protein; and that by % total mass was 6.2% sugar, 31.9% starch, 5% fat, and 23.9% protein. To humans, this chow had a green/brown color and strong odor (likely stemming from ingredients such as fish meal, dried Brewer’s yeast, cane molasses, and meat and bone meal).

The purified chow (TD.230470, Purified Teklad Diet, Inotiv, Lafeyette, IN, USA) had an energy density of 3.0 kcal/g. The macronutrient breakdown by % kcal was similar to that of the standard chow: 52.6% carbohydrate, 15.6% fat, and 31.8% protein; and that by % total mass was 0.4% sugar, 12% maltodextrin, 17% starch, 5.3% fat, and 24.2% protein. To humans, this diet had a white color and a nondetectable odor (likely reflecting the presence of relatively pure nutrients such as corn starch, soy protein, soy oil, casein, maltodextrin and sucrose).

### 2.3. Measurement of Blood Glucose and Plasma Insulin

Blood samples were collected from the tip of the tail, after clipping off the distal 1–2 mm. During sampling, mice were allowed to explore the lid of a mouse cage while the tail was gently milked for blood.

Blood samples were analyzed for plasma glucose and insulin levels. For blood glucose, we analyzed a single drop of blood with a hand-held glucometer (OneTouch Ultra; Milpitas, CA, USA). This brand of glucometer generates blood glucose levels that closely match laboratory biochemical tests [19]. For plasma insulin, we collected ~30 µL of blood in an EDTA-coated capillary tube (Innovative Medical Technologies; Shawnee Mission, KS, USA) and stored it on ice until centrifugation at 4 °C. The decanted plasma was stored at −80 °C until analysis with the Ultra-Sensitive Mouse Insulin ELISA (Crystal Chem; Downers Grover, IL, USA). This test has a limit of detection of 0.05 ng/mL.

### 2.4. Attempt to Replicate Prior CPIR Results with Familiar Lab Chow (Experiment 1)

This experiment sought to replicate a prior report that a single bite from a familiar chow pellet triggers cephalic insulin release in B6 mice [18]. All mice were raised on and tested with standard chow.

We used two experimental designs: between-subjects and matched pairs. In both designs, the mice were food-deprived for 14 h prior to testing. This served two purposes. It limited the quantity of food in the gastrointestinal tract during the test and motivated the mice to initiate chewing.

#### 2.4.1. Between-Subjects Design

The between-subjects design closely matched that of Wiedemann et al. [18]. The experimental mice were placed individually in a clean cage with bedding and one standard chow pellet on the cage-bottom. Immediately after the mouse took a single bite from the pellet, a blood sample was taken. The control mice were placed individually in a clean cage without a chow pellet for 65 s, and then a blood sample was taken. The 65 s latency for control mice matched that used by Wiedemann et al.

In this and all subsequent experiments, we set the alpha level at 0.05 and tested each treatment group for normality, using the Shapiro–Wilk test. Because the data consistently violated the normality assumption, we used nonparametric tests and measures of central tendency for all experiments.

In this experiment, we inferred that the chow elicited a CPIR if the experimental mice exhibited higher plasma insulin levels than the control mice, according to a Mann–Whitney U test.

#### 2.4.2. Matched Pairs Design

We compared experimental and control mice according to a matched pairs design. Mice in both treatment levels were matched (i.e., yoked to one another) based on the amount of time they were in the test cage prior to the blood sample. The experimental mouse was placed individually in a clean cage with bedding and one standard chow pellet on the cage bottom. We recorded how long it took for the mouse to take a single bite from the chow pellet. Immediately after the bite, we removed the mouse from the cage and collected blood for glucose and insulin measurements. The yoked control mouse was also placed individually in a clean cage with bedding, but no standard chow pellet was provided. This mouse was left in the cage for the same amount of time it took for its yoked experimental mouse to take a bite from the chow pellet. After this delay, we removed the mouse from the cage and collected blood for glucose and insulin measurements.

We inferred that the standard chow elicited a CPIR if the experimental mice exhibited higher plasma insulin levels than the control mice, according to a Wilcoxon matched-pairs signed-rank test.

### 2.5. CPIR Test (Experiments 2–5)

The following procedure was used to test for CPIR in Experiments 2–5. Prior to the CPIR test, each mouse was housed individually in a new cage with fresh bedding, and food-deprived for 14 h. At the start of the CPIR test, we (i) removed the mouse from the cage and collected baseline blood samples, (ii) placed a chow pellet on the floor of the cage, (iii) returned the mouse to the cage, (iv) allowed it to chew on the pellet for a predetermined amount of time (see below), and (v) then immediately removed the mouse from the cage and took a second blood sample. All tests were conducted with new (or fresh) chow pellets.

All chewing durations were measured with the stopwatch application on an Apple iPhone (Apple, Cupertino, CA, USA), which has a temporal resolution of 0.01 s. The stopwatch was started when the mouse was placed in the cage. Immediately after the mouse took its first bite, a lap time was recorded to mark the onset of chewing. The first bite was defined as when the mouse secured the pellet within its mouth and the observer visually confirmed that chewing had commenced. When mandibular movements ceased, a second lap was recorded to indicate the end of the chewing bout. This procedure involved two people—one observed the chewing responses, and the other controlled the stopwatch and kept track of cumulative amount of time spent chewing.

Because the mice did not typically chew continuously (i.e., they completed a series of short chewing bouts), we recorded the duration of each chewing bout and ended the test once the sum of the chewing bouts reached the designated chewing duration (e.g., 60 s). If the mouse did not reach its designated chewing duration within 300 s, then it was removed from the experiment.

During pilot studies, we determined that when CPIR testing was conducted in a familiar home cage, the mice spent less time exploring the cage prior to initiating chewing on the chow pellet. For this reason, we transferred each mouse to a clean cage prior to the food-deprivation period and conducted the CPIR test in the same “familiar” cage.

Because elevations in blood glucose alone can trigger insulin release from beta cells in the pancreas [20], we tested for changes in both plasma insulin and blood glucose across the CPIR test. We inferred that chewing on a chow pellet elicited a CPIR if there was a rise in plasma insulin but no change in blood glucose.

### 2.6. How Much Chewing Is Required to Elicit a CPIR? (Experiment 2)

This experiment determined the minimum amount of chewing required for a familiar chow pellet to elicit a CPIR. All mice were raised on the standard chow.

We initially collected a blood sample from each mouse. Then, the mouse permitted to chew on the chow pellet for a total of 0 (control), 5, 15, 30 or 60 s. For the control condition (i.e., 0 s of chewing), the test cage contained no chow pellet, and the mouse was allowed to explore the cage for 300 s prior to taking the second blood sample. For the experimental conditions, the mice were placed in the test cage with a chow pellet until they chewed for the designated duration of time.

We compared blood glucose and insulin levels collected both before and after the chewing event. We inferred that the chewing elicited a CPIR if blood insulin (but not glucose) levels rose significantly above baseline, according to a Wilcoxon matched-pairs signed rank test.

### 2.7. Is Conditioning Required for Standard or Purified Chow to Elicit a CPIR? (Experiment 3)

If Pavlovian conditioning is necessary to condition a CPIR the flavor of the standard or purified chow, then we predicted that (i) each chow would elicit a CPIR when it was familiar, but not when it was novel, and (ii) the previously novel chow would elicit a CPIR following 2 or 4 weeks of dietary exposure. We selected these relatively long exposure durations because prior work indicated that 5 days of exposure was sufficient to condition a CPIR to the flavor of a 32% maltodextrin solution but not to the flavor of a 32% sucrose solution [16]. Based on the results from Experiment 2, all mice were allowed to chew on the chow pellet for a total 15 s during the CPIR test. We assigned mice to one of two treatment groups.

In group 1, we reared mice on standard chow, and maintained their parents on standard chow throughout conception, pregnancy and lactation. On day 1 of the experiment, some of the mice were subjected to a CPIR test with standard chow to confirm that it would elicit a CPIR. The remaining mice were subjected to a CPIR test with purified chow to determine whether it would elicit a CPIR. The latter mice were subsequently maintained on purified chow and were subjected to additional CPIR tests with purified chow at the 2- and 4-week time-points (i.e., days 17 and 31) of the experiment.

In group 2, we reared mice on purified chow and maintained their parents on purified chow throughout conception, pregnancy and lactation. On day 1 of the experiment, some of the mice were subjected to a CPIR test with the purified chow to confirm that it would elicit a CPIR. The remaining mice were subjected to a CPIR test with standard chow to determine whether it would elicit a CPIR. The latter mice were subsequently maintained on standard chow and subjected to additional CPIR tests with standard chow at the 2- and 4-week time-points (i.e., days 17 and 31) of the experiment.

We compared blood glucose and plasma insulin levels collected both before and after the 15 s CPIR test, separately for each test day and treatment group, using the Wilcoxon matched-pairs signed-rank test.

### 2.8. Ingestion Rates on Standard Versus Purified Chow (Experiment 4)

In the previous experiment, we found that a conditioned CPIR to one type of chow did not cross-generalize to the other type of chow. To determine whether differences in chow consistency could explain the absence of cross-generalization, we asked whether the mice consumed each type of chow at a similar rate across the 15 s CPIR test. To this end, we measured (a) the amount of time the mouse took to complete 15 s of chewing and (b) the amount of chow obtained across 15 s of chewing.

All mice were reared on standard chow. At the start of the experiment, we transferred half of the mice to purified chow for one week; the other half were maintained on standard chow for a week.

Afterwards, we food-deprived the mice for 14 h. Then, we subjected each mouse to the CPIR test described in Section 2.5, but we did not collect any blood samples. To determine consumption across the CPIR test, we measured changes in weight of each chow pellet on a microbalance (Metler Toledo XSE105, Columbus, OH, USA) to a tenth of a milligram. We compared each dependent measure across the two chows, using the Mann–Whitney U test.

### 2.9. Is Olfaction a Necessary Component of the Conditioned CPIR to Both Types of Chow? (Experiment 5)

This experiment was inspired by the authors’ impression that the standard chow had a strong odor, whereas the purified chow had a nondetectable odor. We hypothesized that the odor of the standard chow was a salient component of the conditioned stimulus for standard chow. If so, then impairing olfaction should eliminate the conditioned CPIR to standard chow but not purified chow.

#### 2.9.1. Experimental Design

**Figure 1:**
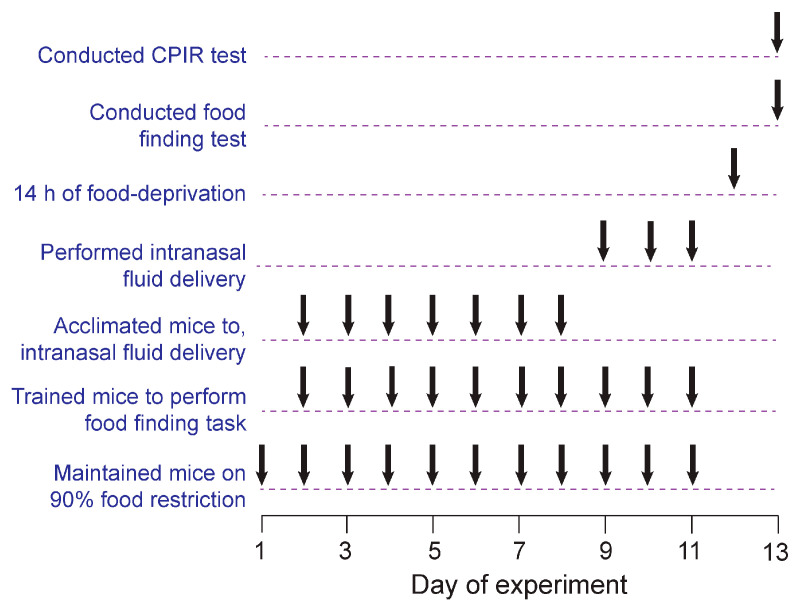
Timeline for Experiment 4. Down-arrows indicate when each of the seven procedures was performed across the 13-day experiment.

Initially, the mice were maintained for at least 4 weeks on standard or purified chow. Afterwards, the mice were subjected to the experimental timeline in Figure 1. On days 1–11, the mice were subjected to 90% food restriction on their familiar chow. On days 2–8, the mice were acclimated to intranasal fluid delivery. On days 2–11, the mice were trained to perform the food-finding task. On days 9–11, the mice were subjected daily to intranasal treatments with the control or ZnSO_4_ solution (see below). On day 12 (14 h prior to the CPIR test) the mice were placed in a clean cage and food-deprived. On day 13, the mice were subjected to the food-finding test in the morning, and the CPIR test in the afternoon.

#### 2.9.2. Method for Impairing Olfaction

The olfactory epithelium of mice was damaged by ZnSO_4_ treatment, using a previously described procedure [21]. First, the mice were acclimated to handling and scruffing and intranasal administration of 10 µL of sterile saline solution on days 2–8 [22] (Figure 1). Next, the mice were subjected to intranasal fluid treatments. A daily treatment consisted of administering a total of 10 µL of a control or experimental solution. The control solution contained sterile isotonic saline + 5% bupivacaine, while the experimental solution contained sterile isotonic saline + 5% ZnSO_4_ + 5% bupivacaine. The bupivacaine was added in to minimize any irritation caused by the ZnSO_4_. During intranasal administration, we pipetted 5 µL of solution onto each nare two times; the mouse subsequently inhaled the solution into the associated nasal cavity. This procedure was repeated across three consecutive days.

To confirm that the ZnSO_4_ treatment impaired olfactory function, we subjected all mice to a food-finding test 24 h after the final intranasal fluid treatment. This test requires an intact olfactory system to be completed successfully [21]. Each mouse had to find a savory snack (Cheeto; Frito-Lay North America, Inc., Plano, TX, USA) buried 3 cm below the surface of fresh bedding (Bed-O-Cobs) in <90 s. To motivate searching for the Cheeto during training, the mice were food-restricted to 90% of their ad libitum body weight [23] (Figure 1).

Prior to the food finding test, the mice were trained individual to locate the Cheeto on days 2–11. On day 2, the mice were given a Cheeto in their home cage, which they consumed avidly. On day 3, they were placed in a new cage with fresh bedding and a partially buried Cheeto; the training session ended when the Cheeto was located. On days 4–11, the mice were placed in a new cage with a Cheeto buried 3 cm under the bedding and given 90 s to find it. All mice learned to locate the buried Cheeto within this 90 s period within 90 s.

On day 13, the mice were subjected to the food-finding task. They were each placed in a new cage with fresh bedding. For a ZnSO_4_-treated mouse to be included in the study, it had to fail the food-finding test—i.e., not find the Cheeto within 90 s of being placed in the test cage. For saline-treated (control) mice to be included, they had to pass the food-finding test—i.e., find the Cheeto within 90 s of being placed in the test cage.

#### 2.9.3. Data Analysis

To test the necessity of olfaction to the conditioned CPIR, we subjected all mice to a 15 s CPIR test with a familiar chow pellet. There were two independent variables: olfactory function (normal or impaired) and type of chow (standard or purified). We made a series of paired comparisons, using the Wilcoxon matched-pairs signed-rank test.

## 3. Results

### 3.1. Attempt to Replicate Prior CPIR Results with Familiar Lab Chow (Experiment 1)

**Figure 2:**
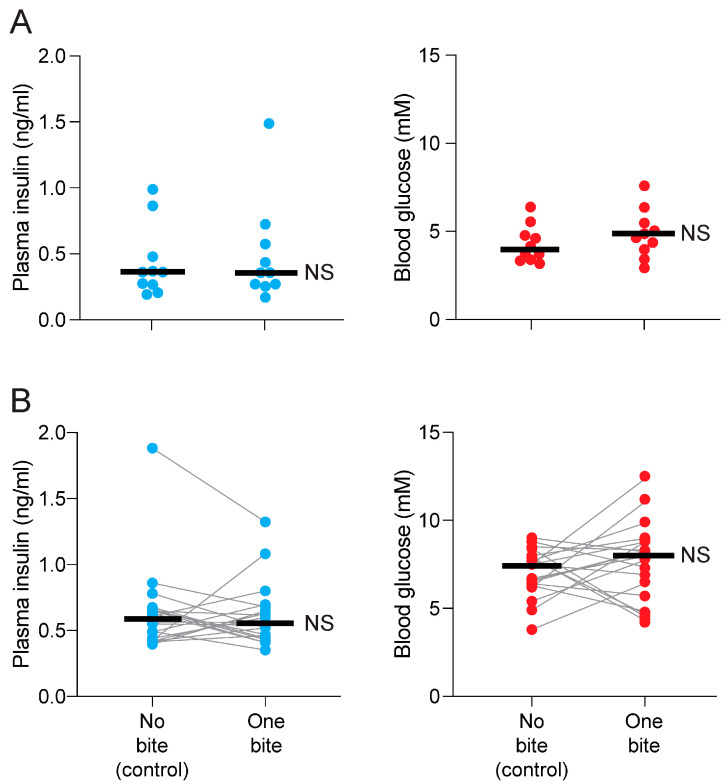
A single bite from standard (familiar) chow did not alter plasma insulin (**left column of panels with blue circles**) or blood glucose (**right column of panels with red circles**) levels in mice (Experiment 1). (**A**) Plasma insulin and blood glucose levels after mice took either no bite (control condition; *n* = 10) or one bite (experimental condition; *n* = 10) from a standard chow pellet. Within each panel, we compare treatment levels with the Mann–Whitney U test; scores from each mouse are indicated with a circle, and median scores with a horizontal line. (**B**) Plasma insulin and blood glucose levels after mice took either no bite (control condition; *n* = 20) or one bite (experimental condition; *n* = 20) from a standard chow pellet. In this latter procedure, each pair of control and experimental mice were matched according to the amount of time they spent in the test cage prior to obtaining the blood samples. Within each panel, we compare treatment levels with the Wilcoxon matched-pairs signed-rank test; scores from each matched pair are indicated with circles connected by a line (NS, *p* > 0.05). Thick horizonal bars indicate median values.

For the between-subjects design, there were no differences in plasma insulin (Mann–Whitney U = 49, *p* = 0.95) or blood glucose (Mann–Whitney U = 37, *p* = 0.34) levels between mice that took a bite from the standard chow pellet (i.e., experimental condition) and those that did not take a bite (i.e., control condition) (Figure 2A).

For the matched-pairs design, we matched mice in each treatment according to the amount of time they were in the cage prior to taking the blood sample; the median latency was 178 s (range: 24–568 s). Despite this matching, there was no difference in plasma insulin (Wilcoxon W = −38; *p* = 0.49) or blood glucose (Wilcoxon W = 59; *p* = 0.28) levels between mice that either did or did not take a bite from the familiar chow (Figure 2B).

These results show that a single bite from a familiar standard chow was not sufficient to elicit a CPIR in B6 mice under our test conditions. Thus, we failed to replicate the CPIR test results reported by Wiedemann et al. [18].

### 3.2. How Much Chewing Is Required for Standard Chow to Elicit a CPIR? (Experiment 2)

**Figure 3:**
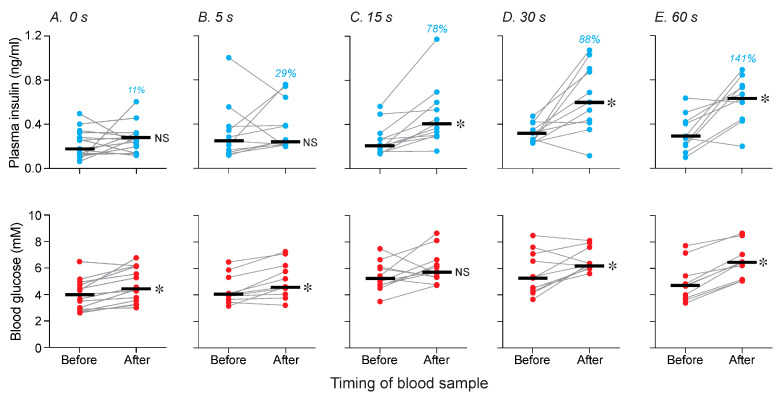
Plasma insulin (**top row of panels with blue circles**) and blood glucose (**bottom row of panels with red circles**) levels varied as a function of total time chewing on a standard chow pellet (Experiment 2). The mice chewed for (**A**) 0, (**B**) 5, (**C**) 15, (**D**) 30 or (**E**) 60 s on the pellet. Within each panel, we show the values taken before and immediately after the mouse completed its designated chewing duration. Scores from each mouse are indicated with circles connected by a line, and medians are indicated with a horizonal line. We indicate the % increase in median plasma insulin in blue text, separately for each panel. We compare the before and after values (within each panel) with a Wilcoxon matched-pairs signed-rank test (NS, *p* > 0.05; * *p* ≤ 0.05). *n* = 10–14 mice/panel.

This experiment asked how much chewing on familiar chow is required to elicit a CPIR. When the mice chewed on standard chow for 0 or 5 s, there were no systematic changes in plasma insulin, relative to the baseline (i.e., “before”) measurement (in both cases, *p* > 0.23; median % increase ≤ 29%) (Figure 3A,B, top row of panels). In contrast, when the mice chewed on standard chow for 15, 30 or 60 s, there was a marked increase in plasma insulin across the CPIR test (in all cases, *p* < 0.01; median % increase ≥ 78%) (Figure 3C–E, top row of panels). Thus, at least 15 s of chewing on standard chow was required to elevate plasma insulin levels.

We also measured changes in blood glucose across the CPIR test. There was a small increase in blood glucose levels following 0, 5, 30 and 60 s of chewing on the familiar chow (Figure 3A,B,D,E; bottom row of panels; in all cases, *p* < 0.032), but not following 15 s of chewing on the same chow (Figure 3C, bottom panel; *p* > 0.10).

Taken together, these results show that a minimum of 15 s of chewing was necessary to elicit a CPIR. Because 15 s of chewing duration was not associated with a rise in blood glucose, we selected this chewing duration for all subsequent CPIR tests.

### 3.3. Is Conditioning Required for Standard and Purified Chows to Elicit a CPIR (Experiment 3)

**Figure 4:**
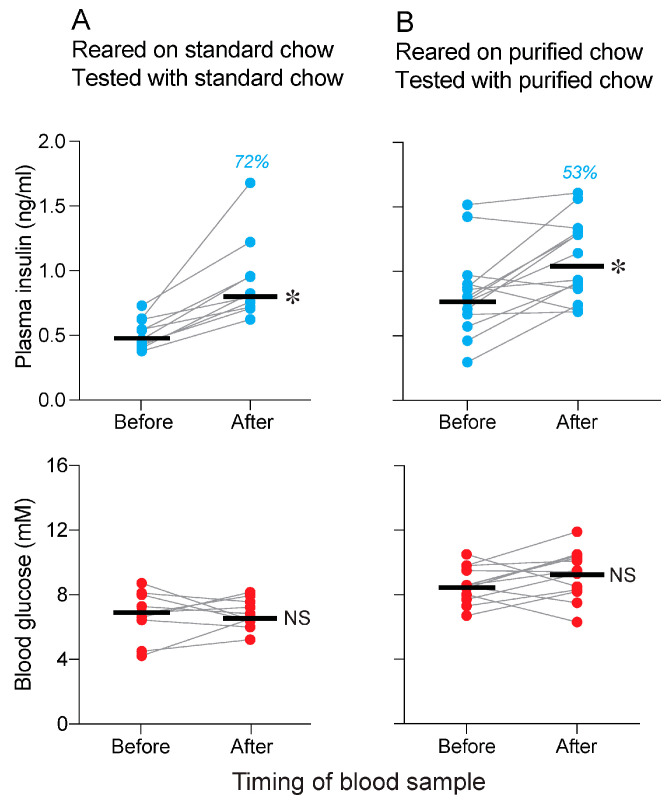
Confirmation that mice exhibit a CPIR to the flavor of (**A**) standard and (**B**) purified chow when it is familiar (Experiment 3). We show plasma insulin (**top row of panels with blue circles**) and blood glucose (**bottom row of panels with red circles**) responses of mice subjected to a 15 s CPIR test. Within each panel, we show values taken before and immediately after the mouse completed 15 s of chewing (i.e., the CPIR test). Scores from each mouse are indicated with circles connected by a line, and median values are indicated with a horizonal line. We indicate the % increase in median plasma insulin in blue text, separately for each panel. We compare the before and after values (within each panel) with the Wilcoxon matched-pairs signed-rank test (NS, *p* > 0.05; * *p* ≤ 0.05). Horizonal bars indicate median values. *n* = 10 mice/treatment group.

We found that 15 s of chewing on standard chow elevated plasma insulin (Wilcoxon value = 55, *p* < 0.002; median % increase = 72%) but not blood glucose (Wilcoxon value = 2, *p* = 0.94) when it was familiar (Figure 4A). We also found that 15 s of chewing on purified chow elevated plasma insulin (Wilcoxon value = 77, *p* < 0.015; median % increase = 53%) but not blood glucose (Wilcoxon value = 10, *p* < 0.96) when it was familiar (Figure 4B).

**Figure 5:**
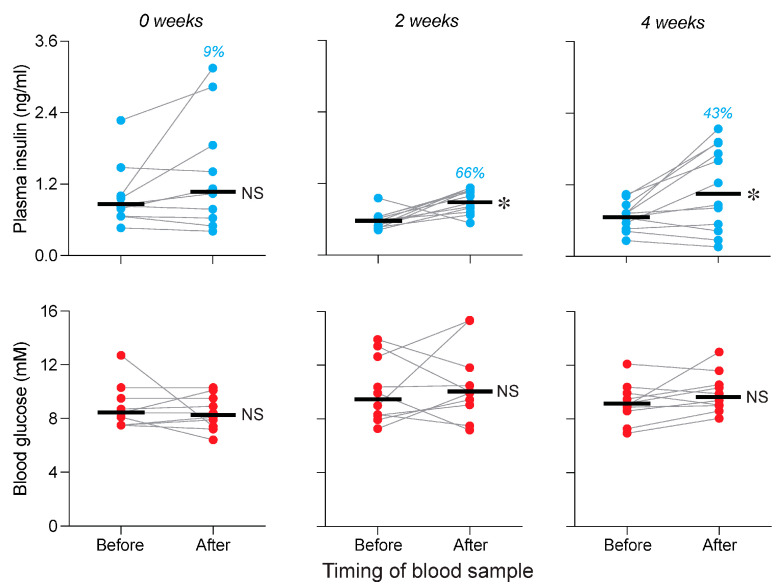
Demonstration that the CPIR to standard chow requires conditioning (Experiment 3). We show plasma insulin (**top row of panels with blue circles**) and blood glucose (**bottom row of panels with red circles**) responses of mice subjected to a 15 s CPIR test. The mice were reared on purified chow and tested with standard chow, following 0, 2 or 4 weeks of exposure to standard chow. The mice with 0 weeks of exposure to standard chow were naïve to it. Within each panel, we show values taken before and immediately after the mouse completed 15 s of chewing. Scores from each mouse are indicated with circles connected by a line, and median values are indicated with a horizonal line. We compare the before and after values (within each panel) with the Wilcoxon matched-pairs signed-rank test (NS, *p* > 0.05; * *p* ≤ 0.05). We tested the same 10 mice after 0, 2 and 4 weeks of exposure to the standard chow.

Next, we asked whether the standard chow would elicit a CPIR when it was novel and if not, whether 2 or 4 weeks of exposure to this chow would condition a CPIR. When the standard chow was novel (i.e., after 0 weeks of exposure), 15 s of chewing chow had no impact on plasma insulin (Wilcoxon W = 25, *p* = 0.23; median % increase = 9%) or blood glucose (Wilcoxon W = −1, *p* = 0.68) levels (Figure 5). We subsequently maintained the same mice on standard chow and re-administered the CPIR test with standard chow at the 2- and 4-week time-points. We found that, at both time-points, 15 s of chewing on standard chow triggered an increase in plasma insulin (Wilcoxon W > 51, *p* < 0.05; median % increase ≥ 43%) but not blood glucose levels (Wilcoxon W < 29, *p* > 0.27) (Figure 5).

**Figure 6:**
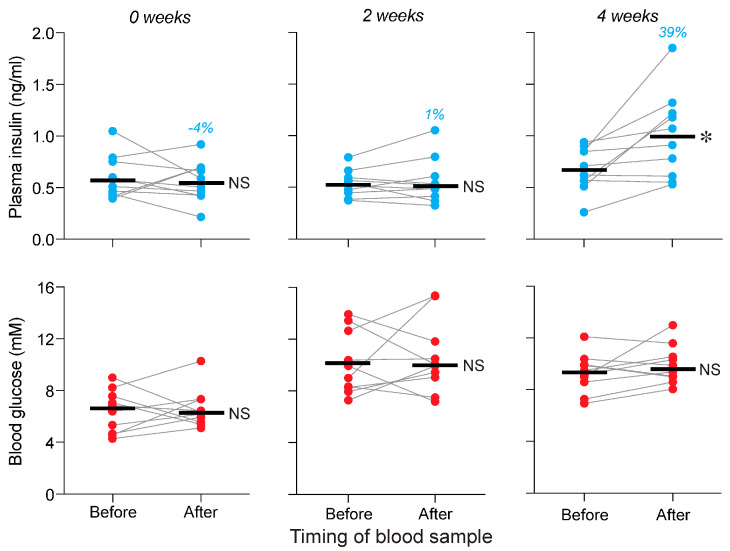
Demonstration that the CPIR to purified chow requires conditioning (Experiment 3). We show plasma insulin (**top row of panels with blue circles**) and blood glucose (**bottom row of panels with red circles**) in mice subjected to a 15 s CPIR test. The mice were reared on standard chow and tested with purified chow, following 0, 2 or 4 weeks of exposure to purified chow. The mice with 0 weeks of exposure to purified chow were naïve to it. Within each panel, we show values taken before and immediately after the mouse completed the 15 s of chewing (i.e., the CPIR test). Scores from each mouse are indicated with circles connected by a line, and median values are indicated with a horizonal line. We indicate the % increase in median plasma insulin level across the CPIR test (in blue text), separately for each panel. We compare the before and after values (within each panel) with the Wilcoxon matched-pairs signed-rank test (NS, *p* > 0.05; * *p* ≤ 0.05). We tested the same 10 mice after 0, 2 and 4 weeks of exposure to purified chow.

For the reciprocal experiment, we asked whether the purified chow would elicit a CPIR when it was novel and if not, whether protracted consumption to this chow would condition a CPIR. We found that 15 s of chewing purified chow had no effect on plasma insulin (Wilcoxon W = −1, *p* > 0.99; median % increase = −4%) or blood glucose (Wilcoxon W = 9, *p* = 0.68) levels across the CPIR test when it was novel (Figure 6). We subsequently maintained the same mice on purified chow and re-administered the CPIR test with purified chow at the 2- and 4-week time-points. We found that 15 s of chewing triggered an increase in plasma insulin at the 4-week (Wilcoxon W < 49, *p* < 0.01; median % increase = 39%) but not the 2-week (Wilcoxon W = 3, *p* = 0.92; median % increase = 1%) time-point (Figure 6). There were no changes in blood glucose levels across the CPIR tests at either of the time-points (Wilcoxon W < 11, *p* > 0.12), but there was an overall increase in glycemia (relative to the start of the experiment).

### 3.4. Ingestion Rates on Standard Versus Purified Chow (Experiment 4)

It took mice an equivalent amount of time to complete 15 s of chewing on both types of chow (Wilcoxon W = 29, *p* = 0.16) (Figure S1A). Further, there was no difference in total amount of chow consumed across the 15 s of chewing (Wilcoxon W = −9, *p* = 0.70) (Figure S1B). The lack of differences in feeding rates on the two types of chow indicate that they had a similar consistency.

### 3.5. Is Olfactory Input a Necessary Component of the Conditioned CPIR Stimulus? (Experiment 5)

**Figure 7:**
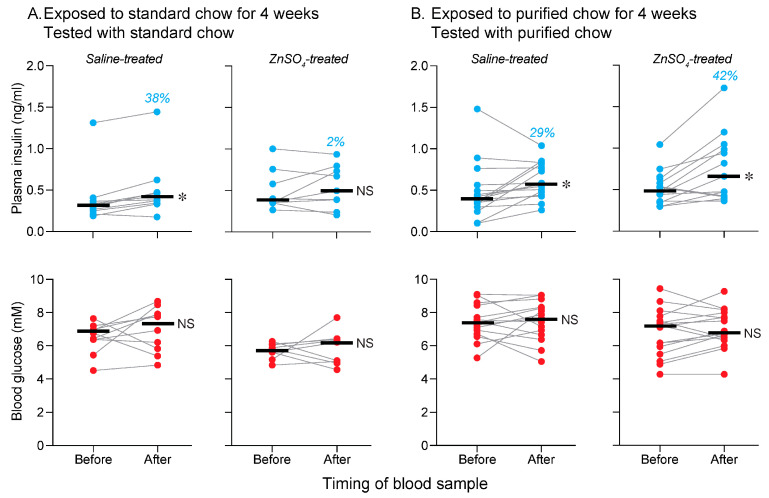
Test of whether olfaction is necessary for the standard or purified chow to elicit a CPIR (Experiment 5). In (**A**), the mice were exposed to standard chow for 4 weeks and then tested with standard chow. In (**B**), the mice were exposed to purified chow for 4 weeks and then tested with purified chow. In both (**A**,**B**), we juxtapose responses of saline- (**left column of panels**) and ZnSO_4_-treated (**right column of panels**) mice. See Figure 1 for experimental timeline. We present levels of plasma insulin (**top row of panels with blue circles**) and blood glucose (**bottom row of panels with red circles**). Within each panel, we show values taken before and immediately after the mouse completed 15 s of chewing (i.e., the CPIR test). Scores from each mouse are indicated with circles connected by a line, and median values are indicated with a horizonal line. We indicate the % increase in median plasma insulin level across the CPIR test (in blue text), separately for each panel. We compare the before and after values (within each panel) with the Wilcoxon matched-pairs signed-rank test (NS, *p* > 0.05; * *p* ≤ 0.05). Horizonal bars indicate median values. *n* = 9–15 mice/treatment group.

In Figure 7A, the mice were exposed to standard chow for 4 weeks, treated with intranasal saline or ZnSO_4_, and then subjected them to the 15 s CPIR test with standard chow. For mice treated with intranasal saline, there was a rise in plasma insulin (Wilcoxon W = 51, *p* < 0.006; median % increase = 38%) but not blood glucose (Wilcoxon W = 23, *p* = 0.27) levels. For mice treated with intranasal ZnSO_4_, there were no changes in plasma insulin (Wilcoxon W = 9, *p* = 0.65; median % increase = 2%) or blood glucose (Wilcoxon W = 3, *p* = 0.91) levels (Figure 7A). In Figure 7B, the mice were exposed to purified chow for 4 weeks, treated with intranasal saline or ZnSO_4_ and then subjected them to the 15 s CPIR test with purified chow. For mice treated with intranasal saline, there was a rise in plasma insulin (Wilcoxon W = 98, *p* < 0.009; median % increase = 29%) but not blood glucose (Wilcoxon W = 10, *p* = 0.80) levels. Likewise, for mice treated with intranasal ZnSO_4_, there was a rise in plasma insulin (Wilcoxon W = 102, *p* < 0.006; median % increase = 42%) but not blood glucose (Wilcoxon W = 38, *p* = 0.30) levels (Figure 7B).

Taken together, these results show that olfaction was a critical component of conditioned CPIR stimulus for standard but not purified chow.

## 4. Discussion

### 4.1. Amount of Chewing Required to Trigger a CPIR in Mice

A single bite from a familiar chow failed to trigger a CPIR in the present study. This negative result was obtained with two different procedures in Experiment 1. In one, we used a between-animal design, and in the other, we used a matched-pairs design, which matched mice according to the amount of time they spent in the cage prior to collecting the blood sample. The latter procedure was implemented to control for any stress associated with time spent in a novel cage. Irrespective of the procedure, there were no differences in plasma insulin or blood glucose levels between mice that took a single bite from the familiar chow and those that did not. In contrast, Wiedemann et al. [18] reported that one bite from a familiar chow pellet was sufficient to elicit a CPIR in mice. It is important to note that we replicated most of the experimental methods used by Wiedemann et al. [18], but there were three notable differences: (i) we used C57BL/6 mice from The Jackson Laboratory (USA), whereas Wiedemann et al. used C57BL/6 mice from Charles River (Germany); (ii) we collected blood from a snipped tail tip, whereas Wiedemann et al. collected blood from a nick of the lateral tail vein; and (iii) we collected blood while the mouse was exploring the cage lid, whereas Wiedemann et al. did so while the mouse was restrained in a slotted tube. These differences could have impacted the amount of stress the mice experienced and thus contributed to the divergent results.

In Experiment 2, we asked whether chewing on a familiar chow pellet triggered a CPIR. To this end, we systematically varied the duration of chewing. We found that 0 (control condition) or 5 s of chewing on the pellet did not increase plasma insulin levels, but 15, 30 or 60 s of chewing did so reliably. Thus, a minimum of 15 s of chewing was required to trigger a rise in plasma insulin. Given that a rise in blood glucose levels alone can stimulate insulin release [20,24], interpretation of the insulin results was complicated by the associated elevations in blood glucose after 0, 5, 30 and 60 s of chewing. Two observations indicate, however, that the elevations in blood glucose did not trigger the elevations in plasma insulin. First, following 0 or 5 s of chewing on the familiar chow, there was a rise in blood glucose but no rise in plasma insulin. We have reported similar findings previously, where consumption of various carbohydrate solutions increased blood glucose but not plasma insulin levels across a 5 min CPIR test [16,25,26]. Second, 30 or 60 s of chewing caused a rise in both blood glucose and plasma insulin levels, whereas 15 s of chewing merely caused a rise in plasma insulin. Taken together, these observations indicate that elevations in blood glucose levels varied independently of elevations in plasma insulin levels across the CPIR tests spanning 0 to 60 s.

To explain the observed elevations in blood glucose levels, we considered the possibility that they were induced by stress associated with the test conditions, given prior reports that human handling can increase blood glucose levels in mice [27,28]. Indeed, our method of sampling tail blood involved human handling. This stress hypothesis, however, does not explain why blood glucose levels increased following 0, 5, 30 and 60 s of chewing in Experiment 2, but not following 15 s of chewing in Experiments 2, 3 and 5. We used the same blood collection procedure in all cases. It follows that stress does not provide a compelling explanation for the observed elevations in blood glucose levels.

In sum, our results demonstrate that 15 s of chewing on standard chow provides a robust measure of CPIR in mice.

### 4.2. The Chow-Induced Cephalic Insulin Responses Required Conditioning

Several investigators established previously that consumption of chow elicits a CPIR in rats [1,17], but they did not explicitly determine whether the CPIR reflected either an unconditioned response to the chow’s flavor or a conditioned response based on prior dietary experience. Here, we present direct evidence that the chow-induced CPIR in mice requires conditioning. When the standard or purified chows were novel, they failed to elicit a CPIR. When both types of chow were rendered familiar by chronic exposure, they elicited a CPIR.

The conditioned CPIR to standard chow appeared after two weeks of exposure, whereas that to purified chow appeared after four weeks of exposure. This observation is consistent with prior reports of variation in the amount of conditioning needed to establish a cephalic response to a food stimulus. We can offer two non-mutually exclusive explanations. One is that the postoral nutritive actions of the standard chow may have more effectively supported the conditioning process. In support of this possibility, we reported elsewhere that a solution of 32% maltodextrin, but not 16% maltodextrin, conditioned a CPIR to the flavor of the maltodextrin solution across five 23 h conditioning sessions. We attributed this finding to differences in the postoral actions of the two maltodextrin solutions [16]. Another explanation stems from the observation that standard chow appears to have a more salient odor to the mice, given that impairing olfaction eliminated the conditioned CPIR to standard chow but not purified chow. Accordingly, a stronger odor (and hence, flavor) intensity of the standard chow may have accelerated the conditioning process (e.g., see [29]).

There is considerable variation in the literature regarding how long it takes to condition a response to a food-related stimulus. For example, it often took > 100 conditioning trials for Ivan Pavlov to condition salivation to an arbitrary sound stimulus in dogs [30], whereas it took Steven Woods only two trials to condition a hypoglycemic response [31] or 21 trials to condition a CPIR [13] to a strong odor stimulus in rats. It took ten 8 min trials for Montaner et al. [15] to condition a CPIR to a peanut butter odor in mice. Recently, we reported that B6 mice conditioned a CPIR to the flavor of a 32% maltodextrin solution after five 23 h conditioning sessions but not after five 1 h conditioning sessions [16]. The present study indicates that it took > 2 weeks to condition a CPIR to the purified chow. Although we did not determine the exact time-course of the CPIR conditioning for standard chow, it certainly appeared within 2 weeks of exposure.

The conditioned CPIR to one type of chow did not generalize to the other type of chow, indicating that each of the chows had unique a flavor profile. Several factors may have contributed to the different flavor profiles. To test for a role of chow consistency, we compared feeding rates on both types of chow. The absence of any difference in feeding rate contradicted the role of chow consistency. We cannot exclude the possibility, however, that the mice perceived more subtle differences in grittiness or texture between the chows during mastication. To test for the role of olfaction, we examined the impact of ZnSO_4_ treatment on conditioned CPIRs to each type of chow. The fact that ZnSO_4_ treatment eliminated the CPIR to standard but not purified chow indicates that olfaction was a salient feature of the conditioned stimulus for standard chow alone. It follows that olfaction could have contributed to the distinct flavor profiles of the two chows. Finally, it is possible that differences in the taste and/or color of the two types of chow could have contributed to divergent flavor profiles. For example, given that each type of chow had a different color, and that the experiments were conducted in a well-illuminated room, the mice could have used differences in chromaticity or luminance to distinguish each type of chow (e.g., see [32]).

### 4.3. Strengths and Limitations of Study Design

The strengths of our study design include the use of negative control treatments and within-subject designs whenever possible. We also replicated several key findings: (i) a single bite from a familiar standard chow pellet did not elicit a CPIR (Figure 2A,B); (ii) 15 s of chewing on a familiar standard or purified chow pellet elicited a CPIR (Figure 3, Figure 4 and Figure 7); and (ii) when a standard or purified chow pellet was novel, it did not elicit a CPIR (Figure 5 and Figure 6). We also documented that the cephalic insulin responses were not confounded by any changes in blood glucose. In Experiment 3, we ensured that each type of chow was novel by both rearing the mice on one type of chow and maintaining their parents on the same chow throughout conception, pregnancy and lactation. This procedure minimized any potential confounding caused by transgenerational epigenetic effects [33]. The external validity of our study was enhanced by using laboratory chows as the conditioning stimulus because these chows model the sensory and nutritional complexity of real foods more effectively than pure carbohydrate solutions.

Our experimental design had several limitations. We limited the design to two types of dry mouse chow. It would be valuable to study a wider variety of dry and wet foods. The distinct composition of the standard and synthetic chows prevented us from identifying the specific orosensory and/or postoral actions of each chow that contributed to the conditioning process. While we identified olfaction as a critical flavor component of standard chow, we did not identify a critical flavor component of purified chow. We determined that >2 weeks of exposure was necessary to condition a CPIR to purified chow, but additional studies are needed to determine how much exposure to standard chow is necessary to condition a CPIR. We also noticed that ≥ 2 weeks of exposure to purified (but not standard) chow increased resting glycemia levels in the mice. Importantly, the elevated glycemia levels did not appear to impact CPIR testing. Finally, we demonstrated that the mice conditioned robust CPIR to each type of diet, but we did not determine how these conditioned CPIRs impacted glucose tolerance or longer-term metabolic parameters.

## 5. Conclusions

To generate cephalic insulin responses to pure solutions of glucose or glucose-containing carbohydrates, mice [34] and rats [35] do not require any prior exposure. In contrast, to generate cephalic insulin responses to the flavor of a laboratory chow (present study) or maltodextrin solution [16], mice require extensive prior exposure to each of these stimuli. It thus appears that mice can trigger cephalic insulin responses through at least two distinct mechanisms. One involves an unlearned reflexive mechanism and the other a protracted conditioning process. The benefit of the latter mechanism is that, at least in theory, it can be conditioned to a wider variety of foods, not just ones with a high concentration of free glucose or glucose-containing carbohydrates.

The conditioned CPIR to each type of chow failed to cross-generalize. If results from these chows are relevant to other types of food, then it follows that mice would have to condition a separate CPIR to each novel food item they encounter, unless the flavor profiles of the two diets overlapped sufficiently. For example, we found that olfaction was a critical component of the conditioned stimulus for the standard diet. It is possible, therefore, that the conditioned CPIR to standard chow would generalize to other types of chow with a similar olfactory signature.

Finally, we limited the focus of this study to cephalic insulin responses. It is likely that these responses constitute just one of many cephalic-phase responses (CPRs) to foods. Indeed, mammals generate a large number of CPRs both immediately before and during a meal to limit disruptions to the internal milieu [36,37]. At this point, however, little is known about the conditions that trigger most CPRs. If they require conditioning, then it follows that there could be a metabolic cost to consuming novel foods. For example, international travel frequently involves the consumption of novel foods. If these novel foods do not elicit CPRs, then this may help explain the high incidence of digestive and metabolic complications in international travelers [38].

## Data Availability

The original contributions presented in this study are included in the article/Supplementary Materials. Further inquiries can be directed to the corresponding author.

## Supplementary Materials

The following supporting information can be downloaded at: https://www.mdpi.com/article/10.3390/nu17243880/s1, Figure S1: Ingestive responses of mice to standard and purified chow (Experiment 4).

## Abbreviations

The following abbreviations are used in this manuscript:

B6 mice	C57BL/6 mice
CPR	cephalic-phase response
CPIR	cephalic-phase insulin response
