# Supplementary material for: Mice Condition Cephalic Insulin Responses to the Flavor of Different Laboratory Chows
Source: Nutrients. 2025 Dec 12;17(24):3880. doi: 10.3390/nu17243880 (PMC12735478; doi:10.3390/nu17243880)
Supplement: Supplementary file 1 [file nutrients-17-03880-s001.zip › nutrients-3990900-supplementary.pdf]

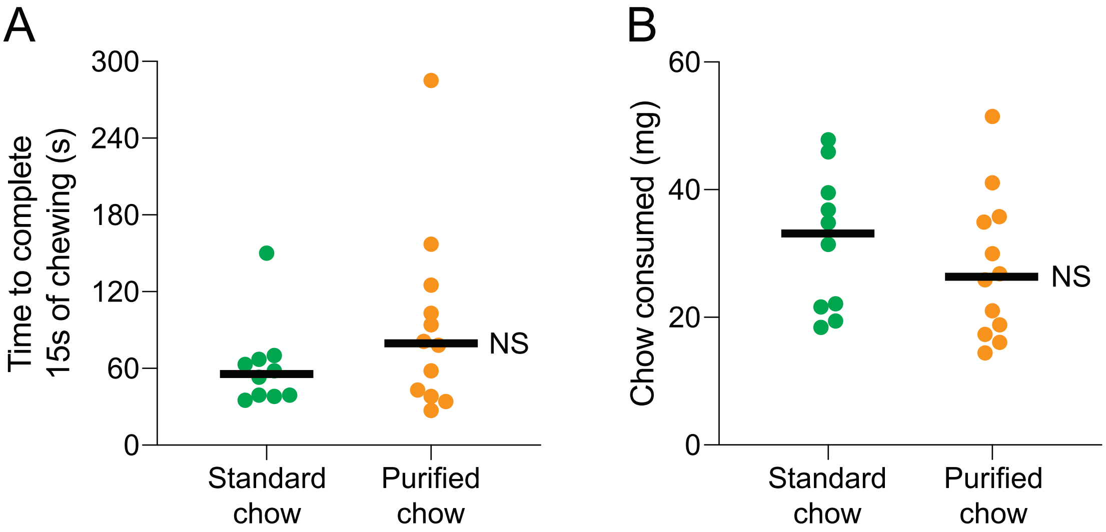

Figure S1. Ingestive responses of mice to standard and purified chow (Experiment 4). For each chow, we show (A) the total amount of time it took for mice to complete 15 s of chewing, and (B) and the amount (in mg) of chow ingested across the 15 s of chewing. Within each panel, we indicate the response of each mouse with a circle, and the median (for each chow) with a horizontal line. We compare values within each panel with the Mann-Whitney U test (NS, $P > 0.05$; $P \leq 0.05$). N = 10-12 mice/panel.
